# A Performance-Based Test for Mitigating the Risk of Geopolymer Concrete Surface Efflorescence Due to Alkali Leaching

**DOI:** 10.3390/ma17153647

**Published:** 2024-07-24

**Authors:** Mahdi Babaee, Arnaud Castel

**Affiliations:** 1 WSP, Sydney, NSW 2000, Australia; 2 Centre for Infrastructure Engineering and Safety, School of Civil and Environmental Engineering, The University of New South Wales, Sydney, NSW 2052, Australia; 3 School of Civil and Environmental Engineering, University of Technology Sydney (UTS), Ultimo, NSW 2007, Australia

**Keywords:** fly ash, GGBS, geopolymer concrete, ion leaching, efflorescence

## Abstract

Geopolymer concretes are considered to be a potential sustainable, low-embodied carbon alternative for Ordinary Portland Cement (OPC) concrete. Alkali leaching is considered to be a major esthetic concern for Na-silicate-based geopolymers as it can lead to the formation of efflorescence products on the surfaces of concrete members exposed to humidity. In this context, this research aims to investigate the effect of the alkali content and the FA/GGBS mass ratio on the alkali leaching and formation of the efflorescence products. Paste cylinders were fabricated and cured in ambient conditions. Samples were submerged in deionized water and the concentration of the leached-out ions was measured. Efflorescence potential was also investigated by partial immersion of the samples in deionized water. The results highlight the complexity of the interacting parameters governing the formation of efflorescence products in geopolymer materials. Establishing relationships between the concrete mix variables and the risk of efflorescence seems unfeasible particularly because of the wide range of possible precursors and activators available to design geopolymer concrete mixes. To overcome this barrier, a practical performance-based testing method is developed. For the first time, by testing a wide range of geopolymer materials, performance-based requirements associated with the risk of efflorescence for geopolymer concrete surfaces exposed to humidity are calibrated. Four categories of risk are proposed and typical suitable exposure conditions for geopolymer concrete surfaces are suggested for each risk category.

## 1. Introduction

Ordinary Portland Cement (OPC) concrete has been used as a building material worldwide for a very long time. However, the use of OPC concrete has been assessed over its lifespan with a particular critique of its energy-intensive and carbon-intensive production process in conjunction with the mass natural resources consumption it requires. As a result, it is urgent that we identify alternative low carbon options [1,2]. One of the alternatives which has been in the forefront of academic research and has appealed to the concrete industry is alkali-activated materials, which is Portland Cement free.

Metakaolin, fly ash (FA) and/or ground granulated blast furnace slag (GGBS) are the most common precursors which are mixed with activator solutions of different alkalinity to produce hardened binders. The main binding phase formed through the alkali activation of aluminosilicate-dominated precursors (metakaolin and FA) is a highly amorphous and cross-linked alkali aluminosilicate-type gel, also known as a “geopolymer” [3,4], whereas in the alkali activation of the calcium-rich GGBS, an alkali charge-balanced aluminum-substituted calcium silicate hydrate is the main reaction product [5,6,7,8]. Due to the limited worldwide availability of GGBS, blends of aluminosilicate raw materials and GGBS are frequently used and their properties have been the subject of many types of research [4]. The presence of GGBS in the mix also improves the pore size distribution and reduces the total porosity which is crucial for developing durable binders [9,10]. Many of the technical properties of OPCs are replicated by alkali-activated and geopolymer-type binders [11], particularly properties such as compressive strength, although, the literature is still scant on topics such as durability and loss of pore solution alkalinity due to alkali leaching.

Alkali leaching can also lead to the formation of efflorescence products on the surfaces of concrete members exposed to humidity, which is especially true for Na-silicate based binders [12,13], and is considered as a major esthetic concern and a barrier for alkali-activated concrete’s widespread adoption by the concrete industry. Parameters such as the alkali content, presence of calcium in the binder, curing regime (ambient cured or heat cured), alkali type (Na or K), and the silicate content have been found to affect the alkali leaching and the efflorescence substantially [12,13,14]. Among those variables, the alkali content is of great importance. Reducing the alkali content to minimize the efflorescence and the alkali leaching will, however, lead to a loss of compressive strength. The determination of an optimum alkali content depends on the reactivity of the aluminosilicate precursors, which can vary for different precursor sources. The presence of calcium-rich precursors in the binder is also another source of complexity in establishing the relationship between the alkali content and the amount of leached-out ions as the calcium ions can be incorporated in the C-A-S-H gel geopolymer network which leads to the release of alkalis [11,12]. Xiao et al. [15,16] used calcium sulfoaluminate cement (CSA) as both a reactive alumina source and a shrinkage-reducing agent to improve the strength and durability properties of alkali-activated materials. They reported that a moderate amount of CSA could considerably reduce the leaching of free alkali because of a higher Al content. The charge imbalance induced by Al favors the immobilization of Na. The same authors also highlighted the strong effect of the pore solution’s pH on alkali leaching.

In this context, this research aimed to investigate the effect of the alkali content and the FA/GGBS mass ratio on the alkali leaching and formation of the efflorescence products. The results highlight the complexity of the interacting parameters governing the formation of efflorescence products in geopolymer materials. Establishing relationships between the concrete mix variables and the risk of efflorescence seems unfeasible particularly because of the wide range of possible precursors and activators available for designing geopolymer concrete mixes. To overcome this barrier, a practical performance-based testing method has been developed. For the first time, by testing a wide range of geopolymer materials, performance-based have been calibrated associated with the risk of efflorescence for geopolymer concrete surfaces exposed to humidity. Four categories of risk are proposed and typical suitable exposure conditions for geopolymer concrete surfaces are suggested for each risk category.

Within the range of precursors and activators considered, the proposed recommendation provides guidance allowing to mitigate the risk of geopolymer concrete surface efflorescence due to alkali leaching.

## 2. Experimental Program

### 2.1. Geopolymer Paste Precursors

An experimental investigation was conducted to assess the ion leaching and efflorescence of geopolymer paste samples fabricated from a blend of Class F fly ash and ground granulated blast furnace slag (GGBS). The low-calcium Class F fly ash was sourced from Gladstone Power Station, Queensland, Australia. The GGBS used in the mixes was supplied by Australian Steel Mill Services (ASMS), Port Kembla, New South Wales, Australia.

The chemical composition of the fly ash and slag pre-cursors, determined by X-ray fluorescence (XRF), are provided in Table 1, alongside the measured loss on ignition (LOI). The amorphous content of the precursors was also determined by X-ray diffraction (XRD) analysis utilizing a 5% ZnO spike, and the results are presented in Table 1.

**Figure 1:**
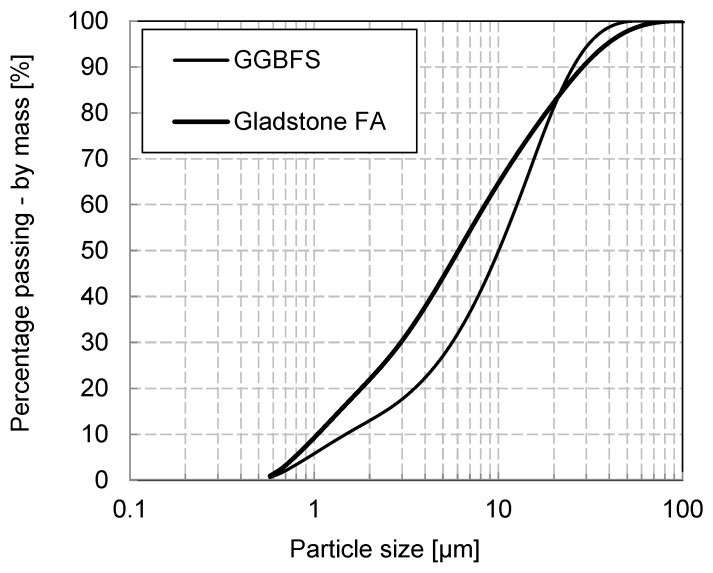
Particle size distribution of FA and GGBS.

The particle size distribution of the aluminosilicate source materials was determined using the laser diffraction technique with a Malvern Mastersizer 2000 instrument (Figure 1). The powders were dispersed in water and sonified before analysis on the instrument.

A mixture of the sodium hydroxide (NaOH) solution and sodium silicate (Na_2_SiO_3_) solution was used. The technical-grade sodium hydroxide pellets with a purity of at least 98% were supplied by Ajax Finechem under the commercial name of UNIVAR A-302. These pellets have a specific gravity of 2.1 g/cm^3^ and a pH of approximately 14. Grade D sodium silicate, which was supplied by PQ Australia under the commercial name of Vistrol D–A53, has a chemical composition of Na_2_O = 14.7%, SiO_2_ = 29.4% and H_2_O = 55.9% (by mass). The Na_2_SiO_3_ solution used is a thick adhesive liquid with a viscosity of 400 cps at 20 °C, has a specific gravity of 1.53 g/cm^3^ and a pH of 12.9 (values provided by the supplier, PQAustralia, Dandenong Victoria).

### 2.2. Geopolymer Paste Mixes and Fabrication

Table 2 presents the details of the geopolymer paste mixes, along with the activator constituents. The concentration of the activators was selected as 4 and 8% Na_2_O by mass of the dry binders (FA + GGBS). The mix proportions were chosen after a set of trial mixes to maximize the compressive strength at a minimum sodium oxide level while having reasonable setting times and workability for mixes 1 and 2. Also, the third mix with a considerably lower compressive strength was considered to assess the proportionality of the concentration of the leaching sodium ions with the sodium oxide level introduced to the system by the alkaline solution.

The NaOH pellets and the Na_2_SiO_3_ solution were mixed in proportions to provide M_s_ (molar ratio of SiO_2_ to Na_2_O) of 1.5, and were allowed to cool down for 24 h before use. The binders were mixed in FA/GGBS proportions of 25%/75% and 75%/25%. The water-to-binder ratio for the mix with 75% GGBS increased as the calcium in the mix consumed the water during the hydration process to form calcium aluminosilicate hydrates (C-A-S-H) after reacting with the silicates and aluminates. The water contents of the NaOH pellets and the Na_2_SiO_3_ solution were considered in the formulation of the solutions and calculation of the water-to-binder ratios. After casting, all samples were cured in sealed molds and were stored in a room with a fixed temperature of 23 ± 2 °C until the testing dates.

### 2.3. SEM-EDS Analysis

Microstructural analysis was performed using a Hitachi S-3400N scanning electron microscope (SEM). Specimens were cold mounted in an epoxy resin and were polished using consecutively finer sandpaper before final preparation using 3-micron and 1-micron diamond pastes on cloths. Specimens were gold and carbon coated for the SEM and EDS tests, respectively. A Quantax 400 energy-dispersive X-ray spectrometer (EDS) was also coupled with the SEM to determine the chemical/elemental composition. Esprit 1.9 software was used to perform the EDS analysis. The accelerating voltage used was 20 kV, and the working distance was set at 10 mm.

### 2.4. Ion Leaching and ICP Analysis

**Figure 2:**
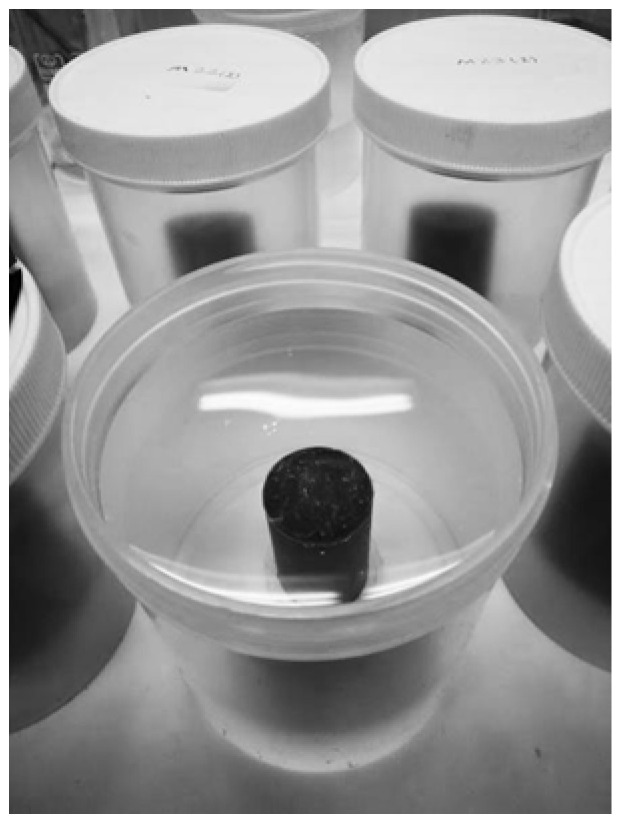
Ion leaching test.

Small paste cylinders (50(H) × 25(D) mm) were fabricated for ion leaching analysis (Figure 2). Samples were submerged in Milli-Q deionized water, and the concentrations of the leached-out ions were analyzed for Na content by inductively coupled plasma-optical emission spectrometer (ICP-OES), using Optima7300DV- ICP-OES (Perkin Elmer, Waltham, MA, USA). The top and bottom surfaces of each sample were sealed using a silicon sealant to eliminate the end effects. The ratio of the total exposed side surface area to the exposure water volume was 100 cm^2^/L for all the samples.

### 2.5. Efflorescence Tests

To investigate the efflorescence due to the leaching of the sodium ions and their reaction with the carbon dioxide in the ambient air, paste cylinders (100(H) × 50(D) mm) were fabricated and were kept in contact with the Milli-Q deionized water at the bottom. The water depth was maintained at 1 to 3 mm constantly. This method has been successfully used previously to accelerate the efflorescence rate [13]. Samples were stored in ambient conditions in a controlled temperature of 23 ± 2 °C and the formation of the efflorescence products was photographed at different stages. After 90 days, white efflorescence products formed on the side and top surfaces of the samples were scraped into a container and weighed using a milligram-precision scale. The density of the efflorescence products was determined by dividing the mass of the scraped efflorescence products in milligrams by the total volume of the sample in cubic centimeters (mg/cm^3^).

## 3. Results and Discussion

### 3.1. SEM/EDS Results

Table 3 shows the EDS analysis results and Table 4 shows the 90 days compressive strength results of the three geopolymer materials tested. Fly ash-based geopolymer binders are X-ray amorphous alkali aluminosilicate gels [17] or geopolymeric micelles [18] with similar alkali binding properties to metakaolin-based geopolymers; i.e., the alkali cations’ incorporation into the geopolymeric network is only possible via a charge balancing mechanism that can offset the charge imbalance of Al^3+^ in the 4-fold coordination [3,19]. On the other hand, slag-based (calcium-rich) systems, mainly comprise a calcium silicate hydrate (C-S-H) gel [6,7,8]. For blended fly ash and slag binders, the co-existence of the aluminosilicate (geopolymeric) gel and the C-S-H gel is proposed, although the formation of C-S-H or the aluminosilicate gel depends on the alkalinity of the alkaline solution and the amount of slag available in the structure [20].

**Figure 3:**
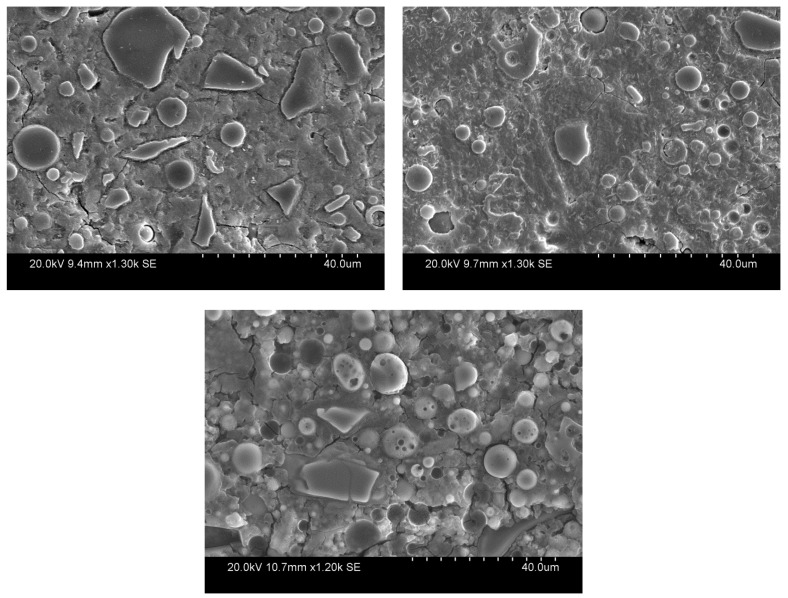
SEM images of Mix 1 (**top left**), Mix 2 (**top right**), and the Mix 3 (**bottom**).

Figure 3 illustrates the SEM micrographs of the mixes mentioned in Table 1. Mix 2 (25% Slag–75% FA) displays a more homogeneous binding matrix compared to the other mixes, which is due to the fact that it has a well-developed geopolymer matrix in a highly alkaline environment. On the other hand, Mix 3 contains undissolved/unreacted FA and slag particles and a more heterogeneous structure. This can be explained by the lack of a high enough alkalinity of the solution, which is required to dissolve the solid aluminosilicate source and produce aluminate and silicate species [17]. Furthermore, the lowest Si/Al ratio was recorded for Mix 3 which is consistent with the compressive strength results (Table 3); Si—O—Si bonds have a higher strength compared to Al—O—Al and Si—O—Al bonds [21], and a lower Si/Al ratio leads to a lower matrix strength.

Ca/Si ratios of the binders are also presented in Table 3. The average Ca/Si ratio of Mix 1 with the highest percentage of slag does not show a proportional increase compared to the other mixes. During the EDS point analysis of the binder for Mix 3, a few points were recorded with the average Ca/Si ratio of 0.99 (close to one), while the average Ca/Si ratio of the rest of the points was 0.38. As was previously discussed in [20], due to the lower alkalinity of the solution and availability of enough slag, a C-S-H gel has been formed, although some Ca ions have leached out to the aluminosilicate gel and have been absorbed into the geopolymer matrix. These phases were indistinguishable during the SEM analysis and only a limited number of points were recorded which can be considered as the representative of the C-S-H gel; as a result, the average Ca/Si ratio for Mix 3 presented in Table 3 should be interpreted in light of such inconsistency.

### 3.2. ICP Analysis and Efflorescence Results

Previous XRD studies of the efflorescence products show that the efflorescence products are hydrous alkali carbonates [13], and provided that the efflorescence products form on the surface of the sample rather than inside the pores, the efflorescence test provides a rapid and basic tool to study the ion leaching. However, the efflorescence test is more of a qualitative method; by contrast, the leaching test yields more quantitative results in more controlled experimental conditions.

**Figure 4:**
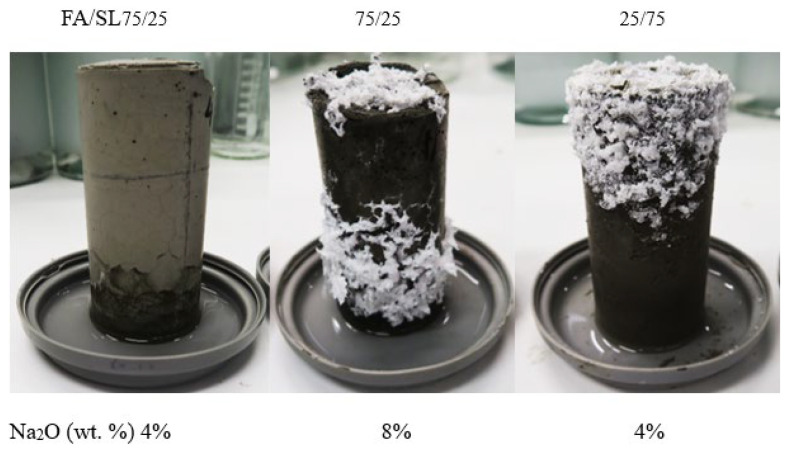
Efflorescence of samples after one month of partial immersion.

Table 5 presents the absolute value of the concentration of the leached-out Na ions, as well as the percentage of the Na ions which have diffused out of the binder. The absolute values are essential for assessing the severity of the efflorescence of the different mixes, while the percentage of the leached-out ions provides valuable information about the relation between the chemical composition and the leaching potential of individual mixes. The percentage of the leached-out ions is calculated as follows:

$$\mathrm{Leached}\;\mathrm{out}\;\mathrm{Na}\;\mathrm{ion}\;(\%)=(1-\frac{{\left[Na\right]}_{L}}{{\left[Na\right]}_{0}})\times 100\;(\%)$$

where [*Na*]*_L_* denotes the amount of the leached-out sodium ions, and [*Na*]_0_ is the total Na content (in the alkaline solution and the amorphous phase of FA and slag). Mix 2 has the highest concentration of leached-out Na ions which is consistent with the efflorescence observations (Figure 4). The absolute values of the concentration of the leached-out ions are not proportional to the amount of sodium oxides introduced into the mixes: the amount of leached-out ions for Mixes 1 and 3 is more than 50% of their Mix 2 counterpart, which is due to the different Ca and Al content of the mixes as well as degree of polymerization. Interestingly, Mixes 1 and 3 have lost their initial sodium content 7.1% and 6.4% more than Mix 2, respectively. The higher-than-expected ion leaching from Mix 1 is in part due to the lower Al content of GGBS compared to FA (Table 1). As mentioned in Section 3.1, tetrahedral aluminum will incorporate the alkali cations in the matrix via a charge balancing mechanism. A reduction in the aluminum content of the binder, as is reflected in the Na/Al ratio presented in Table 3, will increase the availability of the alkali cations in the pore solution. Furthermore, a systematic preference for the incorporation of calcium over monovalent cations into the aluminosilicate gel has been reported [12] from an analysis of the extracted pore solution of a range of mixes with variable FA/GGBS ratios. As is discussed in Section 3.1, Mix 1 is mainly composed of an aluminosilicate (geopolymer) gel with C-S-H as the secondary reaction product. As a result, a small percentage of the calcium is consumed through the hydration process, and the majority of the calcium ions become available to be integrated into the aluminosilicate network which leads to more redundant sodium ions.

Despite having a comparable amount of leached-out sodium ions, Mix 3 did not show any noticeable signs of efflorescence after one month, unlike Mix 1 (Figure 4); this behavior is consistent with the lower Na/Al ratio of Mix 3 (Table 3). The authors hypothesize that although the Na ions are less available to leach out due to the lower Na/Al ratio of the gel and lower Ca content compared to Mix 1, they are more mobile in Mix 3 due to a weak bond; that is, they can easily be dissolved in the water (during the ion leaching test), or they can eventually leach out of the matrix if the efflorescence test would have continued for a longer time (the sample started showing signs of efflorescence after 2 months, but this result is not presented here). It is worth mentioning that the ions can leach out into the solution via two different mechanisms: diffusion due to a concentration gradient and surface dissolution (wash-off). In the case of Mix 3, it seems that the surface dissolution mechanism is the key factor in the unexpectedly high level of the leached-out ions.

### 3.3. Performance-Based Method to Control the Risk of Surface Efflorescence

**Figure 5:**
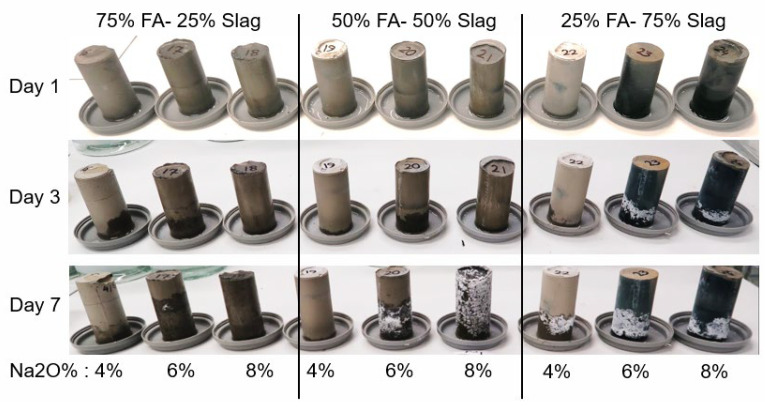
Specimens after 1 to 7 days of exposure.

**Figure 6:**
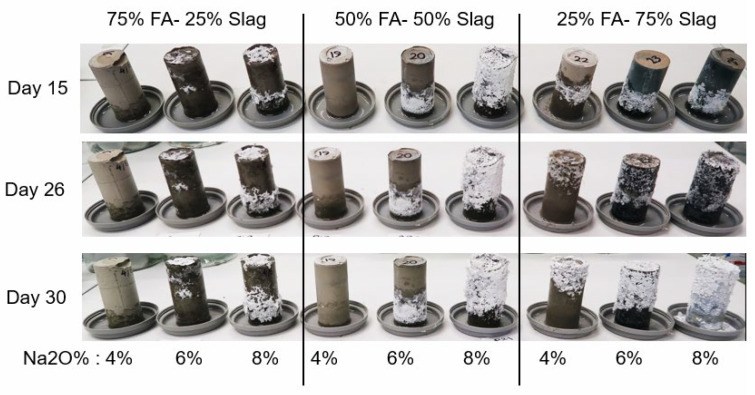
Specimens after 15 to 30 days of exposure.

Considering all the interacting parameters mentioned above, establishing a relationship between the mixes’ variables and the risk of efflorescence seems unfeasible. Thus, a practical performance-based approach was developed to assess the risk of efflorescence based on the amount of leached out ions that reacted with the ambient carbon dioxide to produce white efflorescence products. Paste cylinders (100(H) × 50(D) mm) were fabricated and were kept in contact with the water at the bottom starting 28 days after casting. The water depth was maintained constant at 1 to 3 mm. During the test period, samples were stored in a controlled temperature of 23 ± 2 °C and 50% relative humidity for 90 days before the white efflorescence products formed on the side and top surfaces of the samples were collected. The density of the efflorescence product was determined by dividing the mass of the scraped efflorescence product in milligrams by the total volume of the sample in cubic centimeters (mg/cm^3^). Figure 5 and Figure 6 show photos of the specimens after 1 to 7 days of exposure and after 15 to 30 days of exposure, respectively, showing the time-dependent development of efflorescence products versus mix design parameters. The same geopolymer mixes as the ones shown in Table 2 were tested; in addition, three Na_2_O% were considered for the activator: 4%, 6%, and 8%.

A performance-based guideline was established relating to the risk of efflorescence products appearing on the surface of the geopolymer concrete (Table 6). Four categories of risk are proposed, (1) no risk, (2) low to medium risk, (3) medium to high risk, and (4) high risk, corresponding to a density of scrapped efflorescence products (in mg/cm^3^) of (1) inferior to 1, (2) ranging between 1 and 2.5, (3) ranging between 2.5 and 10, and (4) superior to 10. Table 7 describes the typical exposure conditions suitable (or not suitable) for geopolymer concretes relating to their susceptibility to the creation of efflorescence as per the risk levels proposed in Table 6. The focus is on the likelihood of concrete surfaces to be exposed to humidity during both construction and operational phases. Permanently submerged conditions are not considered in Table 7 as the leaching-out ions would not be exposed to atmospheric carbonation leading to the formation of efflorescence products. These performance-based requirements will assist in selecting suitable geopolymer concrete mixes according to the exposure conditions of the structural member considered.

For each geopolymer paste tested, the test report should include any deviation from the procedure described in this recommendation. The test report should contain the following information: type of precursor, activator, and other relevant information related to mix composition, age and curing history of specimen, and diameter and height of specimen to the nearest 0.5 mm. At least three specimens should be tested for each geopolymer paste mix. The test results shall be reported for each specimen as well as both the average result and standard deviation.

**Figure 7:**
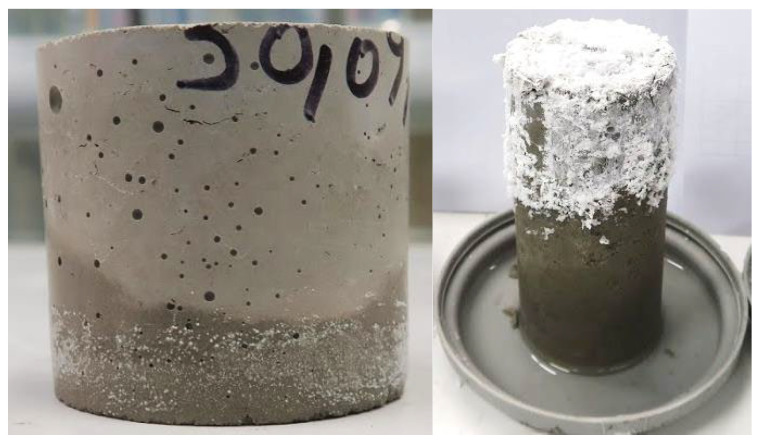
An example of a sample with a medium-to-high risk of efflorescence: concrete sample on the **left** and paste sample on the **right**.

It should be noted that the test method developed was an accelerated approach, and the corresponding concrete sample would experience significantly less efflorescence, as shown in Figure 7. This is due mainly to the different paste content and permeability of concrete samples compared to their paste counterparts.

## 4. Conclusions

Sodium ion leaching and the efflorescence of ambient-cured geopolymer pastes fabricated from blends of fly ash and slag activated using alkaline solution at different concentrations were investigated. The results show that although the alkali content is the most important parameter influencing the ion leaching and the efflorescence formation, the amount of leached-out sodium ions and consequently the severity of efflorescence is not proportional to the alkali content introduced into the mixes. The presence of calcium ions and the degree of geopolymerisation, which depends on both precursor composition and initial alkalinity of the solution, can affect the quantity of leaching ions. This highlights the importance of optimizing the alkali concentration and the calcium content of the mixes to minimize the risk of efflorescence while achieving the desired compressive strength.

Establishing a relationship between the geopolymer mix variables and the risk of efflorescence being extremely complex, controlling the risk of efflorescence through prescriptive requirements relating to the geopolymer mix parameters appeared to be unfeasible. Instead, a performance-based approach was adopted. A practical testing method has been proposed for the first time, aiming to better control the risk of efflorescence formation on geopolymer concrete surfaces. The testing method is simple and only requires basic laboratory equipment. The performance of geopolymer pastes against efflorescence products formation is assessed by measuring the amounts of efflorescence produced at the surface of the specimens following 90 days of controlled exposure to humidity. Four categories of risk are proposed relating to the performance of geopolymer pastes, poorly performing materials being high risk and best-performing materials belonging to the “no risk” category.

Typical exposure conditions suitable (or not suitable) for geopolymer concretes relating to their susceptibility to create efflorescence as per their risk levels are proposed. The focus is on the likelihood of concrete surfaces being exposed to humidity during both construction and operational phases. These performance-based requirements will assist engineers in selecting suitable geopolymer concrete mixes according to the exposure conditions of the structural member considered.

## Figures and Tables

**Table 1:** Chemical compositions of FA and GGBS by X-ray fluorescence (XRF) analysis.

Oxide	Gladstone FA [wt. %]	GGBS [wt. %]
SiO_2_	47.9	35.0
Al_2_O_3_	25.7	14.1
Fe_2_O_3_	14.7	0.36
CaO	4.11	40.9
MgO	1.36	5.51
K_2_O	0.67	0.30
Na_2_O	0.81	0.29
TiO_2_	1.39	0.59
P_2_O_5_	1.21	0.02
Mn_3_O_4_	0.19	0.55
SO_3_	0.19	1.15
Loss of ignition (LOI)	0.69	0.54
Amorphous content	79.1%	100%

**Table 2:** Paste mix designs.

Mix	FA/GGBS	Ms	Na_2_O (wt. %)	Water/Binder *
Mix 1	25%/75%	1.5	4	0.40
Mix 2	75%/25%	1.5	8	0.35
Mix 3	75%/25%	1.5	4	0.35

* Calculated considering the total water and the total solids (precursors + anhydrous activator).

**Table 3:** EDS analysis results.

Mix	FA/Slag	M_s_	Na_2_O (wt. %)	Si/Al (wt. % Ratio) (Molar Ratio)	Na/Al (wt. % Ratio) (Molar Ratio)	Ca/Si (wt. % Ratio) (Molar Ratio)
1	25/75	1.5	4	1.93(1.85)	0.57 (0.67)	0.54 (0.38)
2	75/25	1.5	8	1.94 (1.86)	0.83 (0.97)	0.7 (0.49)
3	75/25	1.5	4	1.71(1.64)	0.41 (0.48)	0.35 (0.245)

**Table 4:** The 90-day Compressive strength of corresponding mortar mixes (50 × 50 × 50 cubic samples).

Mix	FA/Slag	Sand/Binder	M_s_	Na_2_O (wt. %)	Compressive Strength (MPa)
1	25/75	2.75	1.5	4	69.8
2	75/25	2.75	1.5	8	71
3	75/25	2.75	1.5	4	27.5

**Table 5:** Leached-out Na ion concentration after 77 days of immersion.

Mix	FA/Slag	M_s_	Na_2_O%	Leached Out [Na] mM (ppm)	Percentage of Leached Out [Na] Ions (%) (Theory)
1	25/75	1.5	4	31.1 (715)	25.0
2	75/25	1.5	8	40.3 (927)	17.9
3	75/25	1.5	4	31.6 (726)	24.3

**Table 6:** Performance-based guideline to assess the risk of efflorescence.

Density of Scrapped Efflorescence Products (mg/cm^3^)	Risk Level	Example
Density ≤ 1	No risk	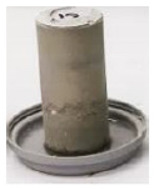
1 < Density ≤ 2.5	Low-to-medium Risk	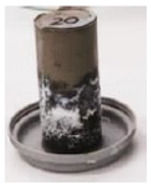
2.5 < Density ≤ 10	Medium-to-high risk	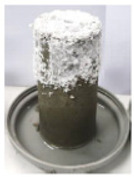
Density > 10	High risk	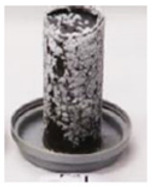

**Table 7:** Risk of efflorescence and typical suitable exposure conditions for geopolymer concrete surfaces in relation to humidity.

Cumulative Density of Efflorescence Products (Weight/Sample Volume) (mg/cm^3^)	Risk of Efflorescence	Guidance on Suitable Concrete Exposure Conditions
<1	Low Risk	Geopolymer concrete suitable for all exposure conditions.
From 1 to 2.5	Low to medium risk	Geopolymer concrete produces only a limited amount of efflorescence if intensively exposed to moisture. Geopolymer concrete suitable for all exposure conditions except Surfaces of members in above-ground exterior environments in areas that are in tropical climatic zone including industrial and non-industrial buildings as well as tidal zone and splash zone and surfaces of members in interior environments in industrial buildings where the member is subjected to repeated wetting and drying.
From 2.5 to 10	Medium to high risk	Geopolymer concrete can produce large amounts of efflorescence if intensively exposed to moisture. Geopolymer concrete can only be used in interior environments, fully enclosed within a residential building except for a brief period of weather exposure during construction.
>10	High risk	Geopolymer concrete is very likely to produce large amounts of fluorescence event if only briefly exposed to moisture. Geopolymer concrete can only be used in interior environments, fully enclosed within a residential building including during construction. Geopolymer concrete should not be used if exposed, even for a brief period, to the external environment during construction.

NOTE: High risk of efflorescence can be suitable for surface of members in contact with the ground if the formation of efflorescence products is not an esthetic issue (i.e., members are buried).

## Data Availability

The original contributions presented in the study are included in the article, further inquiries can be directed to the corresponding author.
